# Therapeutic Potential of Fingolimod and Dimethyl Fumarate in Preclinical Pancreatic Cancer Models

**DOI:** 10.32604/or.2025.072141

**Published:** 2026-02-24

**Authors:** Pauline Gousseau, Laurie Genest, Guillaume Froget, Tristan Rupp

**Affiliations:** Porsolt SAS, ZA de Glatigné, Oncology Group, Le Genest-Saint-Isle, 53940, France

**Keywords:** Pancreatic cancer, preclinical models, tumor progression, fingolimod, dimethyl Fumarate

## Abstract

**Objectives:**

The five-year survival rate for pancreatic cancer is notably low, posing a significant challenge to patient health. The primary treatments are radiotherapy and chemotherapy, sometimes combined with targeted therapy; however, their clinical benefits are limited. Therefore, developing new models to evaluate the therapeutic potential of novel molecules is essential. Fingolimod and Dimethyl Fumarate (DMF), currently used to treat multiple sclerosis, have recently been shown to have anti-cancer effects in several preclinical tumor models. This study aims to evaluate the therapeutic potential of Fingolimod and DMF in pancreatic cancer by investigating their respective *in vitro* cytotoxicity and *in vivo* antitumor effects.

**Methods:**

In this study, we evaluated for the first time these two drugs in pancreatic preclinical models *in vitro* using 3D spheroid tumor models and *in vivo*, which are compared to two standard-of-care consisting of Gemcitabine and Erlotinib.

**Results:**

*In vitro*, both Fingolimod and DMF induced cytotoxicity in spheroids from two pancreatic cell lines. Additionally, Fingolimod and DMF displayed anticancer effects in two subcutaneous xenograft models using PANC-1 and CFPAC-1 cells.

**Conclusions:**

Although the responses observed with Fingolimod and DMF were similar to those of Gemcitabine and Erlotinib, these findings indicate a potential emerging interest in Fingolimod and DMF for the treatment of pancreatic cancer. However, further work is still necessary to fully characterize how these drugs affect tumor progression.

## 1 Introduction

Pancreatic cancer (PC) is a major public health problem worldwide, particularly in developed countries. The 5-year survival rate does not exceed 10% [1]. In 2021, the incidence and mortality rates for PC were 5.96 and 5.94 per 100,000 people, respectively [1]. In 2022, PC ranked as the sixth leading cause of cancer-related mortality, affecting all demographic groups. By 2030, PC is projected to become the second deadliest cancer worldwide, largely due to ineffective treatment options [2]. Common symptoms include abdominal pain, weight loss, nausea, and fatigue [3]. The tumor can also obstruct the bile ducts, which may impair liver function [4]. While PC tumors develop gradually, often delaying diagnosis, the disease progresses rapidly once symptoms appear [3]. The predominant risk factors are behavioral, including smoking, alcoholism, and obesity; meanwhile, genetic factors account for only 10% of cases [5].

Surgery currently offers the best prognosis for patients without metastases [6]. Unfortunately, only 20% of patients are eligible, as most present with metastatic disease at diagnosis [6]. Radiotherapy is typically used post-surgery to reduce tumor recurrence; it can also be administered pre-surgery to aid tumor resection [6]. For unresectable tumors, radiotherapy is often combined with chemotherapy. Gemcitabine is the standard chemotherapeutic treatment of PC for over 20 years [3,7]. After metabolism, Gemcitabine inhibits DNA synthesis and induces cell apoptosis [8]. A primary clinical study has demonstrated that Gemcitabine prolongs median survival to 5.65 months, compared to 4.41 months with 5-fluorouracil (5-FU), and increases the one-year survival rate from 2% to 18% [9]. When combined with the Epidermal Growth Factor Receptor (EGFR) inhibitor Erlotinib, median survival reaches 6.24 months, with a one-year survival rate of 23% [10]. Despite these advances, the clinical benefit remains very limited due to the development of micro-metastases at an early stage of the disease [4]. Thus, there is an urgent need to develop and validate new therapeutic strategies to improve prognosis and patients’ health and quality of life.

Fingolimod and Dimethyl Fumarate (DMF) are drugs approved to treat multiple sclerosis and are well tolerated by patients [11,12]. Fingolimod is a structural analogue of sphingosine-1-phosphate (S1P) that acts as a sphingosine-1-phosphate receptor (S1PR) antagonist [13]. Due to this function, Fingolimod induces the sequestration of lymphocytes in the secondary lymphoid organs, including lymph nodes. Fingolimod has demonstrated anti-tumor effects using syngeneic and xenograft mouse models of triple negative breast cancer [14], non-small cell lung cancer [15], colon cancer [16], prostate cancer [17], liver cancer [18], and ovarian cancer [19]. Conversely, in other tumor indications such as glioblastoma, Fingolimod did not affect tumor growth *in vivo* [20]. Shen et al., 2007 showed *in vitro* that Fingolimod both suppresses cell proliferation and promotes apoptosis [21]. DMF has been shown to act on the signaling pathway mediated by Nuclear factor erythroid 2-related factor 2 (NRF2) and thus influences the cellular response to oxidative stress [22]. DMF at low doses, shows cardioprotective, neuroprotective, and hepatoprotective effects [23–25]. At higher doses, the cytotoxic effects of DMF have been demonstrated on numerous cancer cell lines including ovarian, colon, and lung cancers [26,27]. However, these results are noteworthy, but the effects of Fingolimod and DMF have so far been poorly or not investigated in pancreatic preclinical animal models and relevant 3D culture. In fact, we are aware of only limited literature exploring the effect of Fingolimod on PC and no research has been conducted to assess the impact of DMF. While previous research demonstrated that Fingolimod can induce an anti-tumor effect in *in vitro* 2D monolayer standard culture conditions in a short list of PC cell lines including PANC-1 cells, no one has evaluated yet its effect in a complex 3D *in vitro* models and extensively in an *in vivo* xenograft models [28–32]. Moreover, except for one recent article that evaluated the effect of DMF *in vitro* 2D monolayer standard culture conditions, no study has evaluated the response *in vivo* and *in vitro* complex models [33].

This study was designed to assess the therapeutic potential of Fingolimod and DMF in PC through three complementary levels of investigation: clinical, *in vitro*, and *in vivo* analyses. We first evaluated the expression level in the clinical dataset of S1PR and NRF2, respectively, affected by Fingolimod and DMF. Then, to evaluate the therapeutic potential of Fingolimod and DMF in PC, we characterized their cytotoxicity and anti-tumor effects in complementary preclinical models. Therefore, we performed a systematic phenotypic analysis using two cellular models of PC (PANC-1 and CFPAC-1 human cell lines) upon DMF or Fingolimod treatments, followed by *in vivo* validation in subcutaneous xenograft models. The present study represents the first systematic validation of these drugs specifically in PC models, a malignancy characterized by resistance to conventional therapies and a dismal prognosis. To our knowledge, this work provides: (1) the first evaluation of DMF in both 3D *in vitro* and *in vivo* models of PC; (2) the empirical evaluation of Fingolimod in two new *in vivo* models using PANC-1 and CFPAC-1 cells; and (3) the preliminary safety assessment of Fingolimod and DMF.

## 2 Materials and Methods

### 2.1 Patient Data Analysis

The clinical data depicted herein are compiled from mixed publicly available datasets from The Cancer Genome Atlas (TCGA) and The Genotype-Tissue Expression (GTEx) project, specifically concerning PDAC cohorts [34,35]. Overall survival (OS) was assessed through the Kaplan–Meier method, using the online platform referred to as ‘Kaplan-Meier Plotter’ for the analysis (http://gepia.cancer-pku.cn/ accessed on 15 December 2023) [36]. Patients were categorized into low-expression and high-expression groups based on the median value as the threshold. Furthermore, the expression levels of the targeted genes were analyzed through the online platform ‘Expression DIY-Boxplot’ (http://gepia.cancer-pku.cn/ accessed on 15 December 2023) [36]. The statistical evaluation was performed utilizing a Student *t*-test, with the disease state (Tumor or Normal) serving as a variable for the assessment of differential expression.

### 2.2 Cells and Cell Culture

The cell culture procedure was carried out according to previous work [20]. All the cells were STR authenticated by the provider prior to use. CFPAC-1 is derived from liver metastases from a patient with cystic fibrosis [37] and was obtained from the ATCC (CRL-1918™, Manassas, Virginia, USA). PANC-1 were isolated from a PDAC [38] and were obtained from the ATCC (CRL-1469™). Both cell lines were cultured in RPMI 1640 (reference A10491-01 ATCC-formulated, Gibco^®^ Billings, Montana, USA) supplemented with fetal bovine serum (FBS, reference 10270-106, Gibco^®^) at a final concentration of 10% and including antibiotics (Penicillin 100 U/mL-Streptomycin 100 µg/mL, reference 15140122, Gibco^®^). All procedures were conducted under aseptic conditions as previously described [39]. The cells were maintained at 37°C with a 5% CO_2_ environment. After thawing, the cells were limited to a maximum of 10 passages from the initial stock provided by the ATCC. Testing for mycoplasma demonstrated that all cell lines were negative results using the MycoAlert^®^ Mycoplasma Detection Kit (reference LT07-318, Lonza™, Basel, Switzerland). Cells were allowed to reach a confluence of 70%–90% before being split and prepared at specified concentrations for *in vitro* or *in vivo* assays. The assessment of cell count was conducted using a cell counter (Nucleocounter NC-200™, ChemoMetec^®^ Allerod Denmark).

### 2.3 In Vitro Treatment

Gemcitabine (S1149, Selleckchem™, Cologne, Germany) and Fingolimod (S5002, Selleckchem™) were directly prepared in the culture medium, while Erlotinib (S7786, Selleckchem™) and DMF (S2586, Selleckchem™) were first solubilized in dimethyl sulfoxide (DMSO, 295522500, Acros Organics™, Geel, Belgium) then diluted in culture medium. For concentrations used, see Table 1.

**Table 1:** Treatment concentrations used *in vitro* (in µM)

Treatments	Gemcitabine	Erlotinib	Fingolimod	DMF
**Concentrations (µM)**	0.2, 2, 20, and 200	0.1, 1, 5, and 10	1, 2.5, 10, and 20	5, 25, 50, and 100

### 2.4 3D Tumor Spheroid Assay

The procedure was adapted from previous work [15]. Cells were plated in 96-well CellCarrier Spheroid Ultra Low Adhesive (ULA) Microplates™ (reference 6055334, Perkin Elmer^®^ Shelton, Connecticut, USA) at 2000 cells/well in culture medium. ULA Microplates™ have a U-shaped bottom to limit the adhesion of cells to the plastic and to promote the spontaneous formation of spheroids. After a culture of 48 h, spontaneously generated tumor spheroids were detected usingPANC-1 cells. The culture medium was supplemented with 100 µg/mL of Matrigel^®^ (reference 356237, Corning^®^ New York, USA) to promote the formation of CFPAC-1 spheroids, and tumor spheroids appeared after 48 h of growth. Spheroids were then treated with Gemcitabine, Erlotinib, DMF, or Fingolimod in culture medium at the concentrations described in Table 1. To detect dead cells in spheroids, a fluorescent DNA intercalating agent was added, which stained cells when their plasma membranes were compromised (Sytox™ green Dead Cell Stain, reference S7020, final concentration at 100 nM, ThermoFisher Scientific™, Waltham, Massachusetts, USA). Imaging was performed at four-hour intervals for a total of 96 h. The confluence and the area of positive fluorescence were analyzed through the Incucyte™ system (Sartorius^®^ Goettingen, Germany). Cytotoxicity was defined as a percentage, determined by calculating the fluorescent positive area in pixel^2^ as a proportion of the total spheroid area (in pixel^2^). Each test substance was subjected to three independent experiments, with each experiment including 3 to 4 technical replicates.

### 2.5 Animals

The housing conditions were adapted in accordance with prior work [14]. Female NOD.CB17_Prkcscid/Rj (NOD-SCID) or male BALB/cAnN-Foxn1nu/nu/Rj (BALB/c-nude) mice were obtained from Janvier Labs at 6 weeks old (Le Genest-Saint-Isle, France). Prior to the implantation of tumor cells, the animals underwent an acclimatization period of at least five days, then the procedures were conducted on mice aged of 7 to 8 weeks. A total number of 100 mice were used in this study, 50 female NOD-SCID mice and 50 male nude mice. Mice were housed with up to 6 animals per cage in a biosafety level 2 laboratory in individually ventilated cages (NEXGEN MOUSE IVC™, Allentown^®^, New Jersey USA) with NestPak^®^ (Allentown^®^). Nesting materials, including tubes, cotton, and wood were provided for enrichment. The environment was controlled with artificial lighting on a 12-h cycle from 7 a.m. to 7 p.m., maintaining a temperature of 22 ± 2°C. The mice had unrestricted access to a rodent diet (SAFE^®^ R04-40, Augy, France) and water. Identification of the mice was achieved through permanent tattoos. The number of mice in each experimental group is detailed in the figure legends.

### 2.6 In Vivo Treatments

Erlotinib, Fingolimod, and DMF were diluted in 5% DMSO + 30% polyethylene glycol (PEG) 400 + 5% Tween 80 + H_2_O. Gemcitabine was diluted in physiological saline. Drugs or vehicles were administered five times per week, per os (p.o.), until the end of the experiment, except for Gemcitabine and its vehicle which were administered once a week, via intraperitoneal (i.p.) route (Table 2).

**Table 2:** Treatments used *in vivo*, concentrations and administration route

Groups	Treatments
Control	Vehicle intraperitoneal [i.p.] (once a week) + vehicle per os [p.o.] (five times a week)
Gemcitabine	Gemcitabine 80 mg/kg, i.p. (once a week) + vehicle p.o. (five times a week)
Erlotinib	Vehicle i.p. (once a week) + Erlotinib 50 mg/kg, p.o. (five times a week)
Fingolimod	Vehicle i.p. (once a week) + Fingolimod 5 mg/kg, p.o. (five times a week)
DMF	Vehicle i.p. (once a week) + DMF 30 mg/kg, p.o. (five times a week)

### 2.7 Subcutaneous Xenograft Animal Model

The subcutaneous (s.c.) grafting procedure was adapted from previous work [15]. The cells were resuspended in sterile phosphate-buffered saline (PBS, reference10010023, Gibco^®^) and kept chilled on ice prior to their injection. In the case of PANC-1 cells, 20 × 10^6^ cells were injected into NOD-SCID mice, whereas 5 × 10^6^ CFPAC-1 cells were administered to BALB/c-nude mice, with each injection consisting of 100 µL of the cell suspension using an insulin syringe. Both cell lines were introduced subcutaneously into the right flank of the mice, which were anesthetized with 2% isoflurane (reference 152678, Axience^®^, Pantin, France) at a flow rate of 1.5–3 L/min. Eye lubricant was applied throughout the procedure, and the mice were positioned on a warming pad. The injection area was shaved only for the NOD-SCID mice and was cleaned with Chlorhexidine (Antisept™, reference ANT015, Axience^®^). The mice’s breathing was continuously monitored until they awoke. Tumor volume was assessed using the formula V = (a^2^ × b)/2, where b is the longest axis and a is the axis perpendicular to b. This measurement was taken two to three times weekly with a caliper. Additionally, a range of physiological and behavioral indicators were monitored during the experiment, which included temperature, dyspnea, consumption of food and water, balance issues, and sedation, when required. Prior treatment initiation when the tumors reached an average volume of approximately 100 mm^3^, mice were randomized based on their body weight and tumor volume through the five group treatments and ten mice were allocated per group. Mice received either (1) both i.p. and p.o. vehicles, (2) Gemcitabine 80 mg/kg i.p and p.o. vehicle, (3) vehicle i.p and Erlotinib 50 mg/kg p.o., (4) vehicle i.p and Fingolimod 5 mg/kg p.o., (5) vehicle i.p and DMF 30 mg/kg, p.o. (see Table 2). Mice without palpable tumor were excluded from the study. The study was performed in a non-blind manner.

### 2.8 Animal Ethical Consideration and Limit Points

The approach was adapted in accordance with prior studies and standard internal guideline [15]. The strategies implemented were aimed at minimizing the suffering experienced by animals. The experiments were conducted in strict alignment with Council Directive No. 2010/63/EU, effective from 22 September 2010, and the French decree No. 2013-118 (1 February 2013) regarding the protection of laboratory animals. The experiments were done in a laboratory holding accreditation from the Association for Assessment and Accreditation of Laboratory Animal Care (AAALAC). The animal study was approved by the Internal Animal Care and Use Committee (IACUC) of Porsolt by the French Ministère de l’enseignement supérieur et de la recherche (national agreement n° F 53 1031, authorization project number: 2022100312442012). The following criteria were identified as critical limit points that justified the euthanasia of the mice through CO_2_ inhalation or cervical dislocation including a tumor volume exceeding 2000 mm^3^, a weight loss greater than 20% in relation to the initial weight over two consecutive measurements, severe tumor necrosis or ulceration, hypothermia (below 35°C), respiratory distress, inability to eat or drink, loss of balance, and marked levels of sedation.

### 2.9 Statistical Analysis

Statistical analyses and graphical visualizations were conducted utilizing GraphPad Prism (version 9.4.1 or later, GraphPad Software, San Diego, CA, USA). A *p*-value threshold of less than *p* < 0.05 was considered statistically significant. The Gaussian or normal distribution was tested using the D’Agostino–Pearson test. The analysis of *in vitro* 3D spheroid growth and cytotoxicity was done using a mixed-effects model (restricted maximum likelihood, REML) with repeated measures, incorporating group and day as factors. This was complemented by Bonferroni’s multiple comparison test to compare each day against the control group. Similarly, tumor volume and body weight data were evaluated through a mixed-effects model (REML) with repeated measures, with group and day as factors, followed this time by Tukey’s multiple comparison tests for each day. The tumor weights were analyzed using a one-factor ANOVA (treatment effect) and were compared to the control group using Tukey’s post-hoc test.

## 3 Results

### 3.1 Analysis of Public Clinical Data: Expression of Downstream Target of Fingolimod and DMF Are Upregulated in PC Cohorts

**Figure 1:**
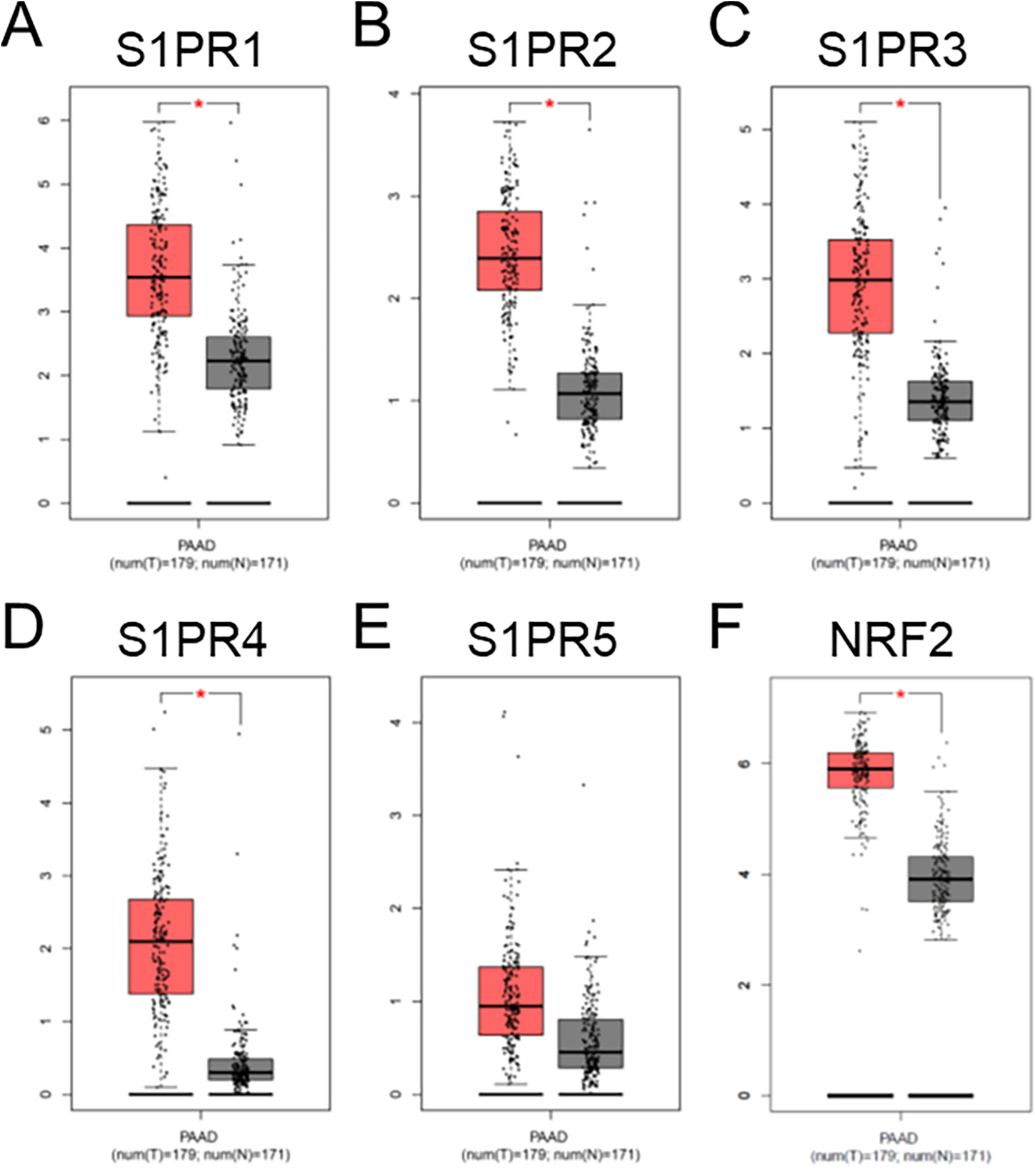
Analysis of publicly available clinical data of S1PR1 (**A**), S1PR2 (**B**), S1PR3 (**C**), S1PR4 (**D**), S1PR5 (**E**), and NRF2 (**F**) expressions in pancreatic cancer patients. Tissues were stratified in tumoral (T, in red, n = 179) and no-tumoral (N, in grey, n = 171) groups. *p < 0.05

We conducted an analysis of publicly accessible datasets relating to patients diagnosed with PC. Our analysis focused on the differential expression of genes in pancreatic adenocarcinoma (PAAD) tissues compared to non-cancerous pancreatic tissue, specifically evaluating S1P signaling receptors, S1PR1, S1PR2, S1PR3, S1PR4, and S1PR5 [40], and DMF’s target, NRF2 [27]. We observed that PAAD tissues displayed higher expression of all five S1PR (S1PR1, S1PR2, S1PR3, S1PR4 and S1PR5) in comparison to normal tissues, with statistically significant increases for S1PR1, S1PR2, S1PR3, and S1PR4 (Fig. 1A–E). Fingolimod primarily targets S1PR1, as well as S1PR3, S1PR4, and S1PR5, but does not affect S1PR2 [41]. Moreover, NRF2 also displayed a significant higher expression compared to normal tissue (Fig. 1F). NRF2 is known to play a critical role in anti-inflammatory responses and has been linked to a reduction in tumor burden [42,43]. These results indicate that the strategy targeting S1P and NRF2 signaling pathways may hold significant therapeutic promise for PC patients, suggesting that Fingolimod and DMF could be promising therapeutic agents.

### 3.2 Fingolimod and DMF Induced In Vitro Pancreatic 3D Spheroid Cytotoxicity

Typically, the use of 2D models is considered a standard approach; however, 3D tumor spheroids provide a marked benefit by more effectively mimicking the complexity and variability found within tumors [44]. The 3D architecture of tumors creates chemical gradients and physical barriers, exposing cells to varying levels of oxygen, nutrients, growth factors, and cell-cell adhesion in the tumor microenvironment (TME), which cannot be fully recapitulated in 2D cultures [44]. Ultra-low attachment (ULA) plates with round-bottom wells facilitate the formation of spheroids of consistent size [45]. In this work, a 3D culture was developed using pancreatic tumor spheroid models to evaluate the effects on cell viability of Fingolimod and DMF, compared to the standard treatments Gemcitabine and Erlotinib [10]. Our initial findings showed that PANC-1 generated cohesive tumor spheroids after 48 h of culture in ULA plate (Fig. S1A), but the CFPAC-1 cells did not (Fig. S1B). Conversely, culturing CFPAC-1 cells in Matrigel^®^-containing medium at 100 µg/mL promoted cellular aggregation (Fig. S1C). Indeed, Matrigel^®^ composed primarily of laminin-111 and collagen IV, supports intercellular interactions [46].

**Figure 2:**
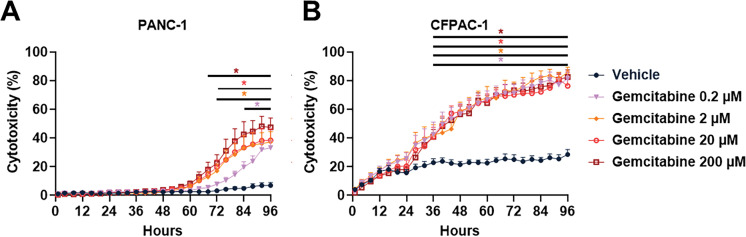

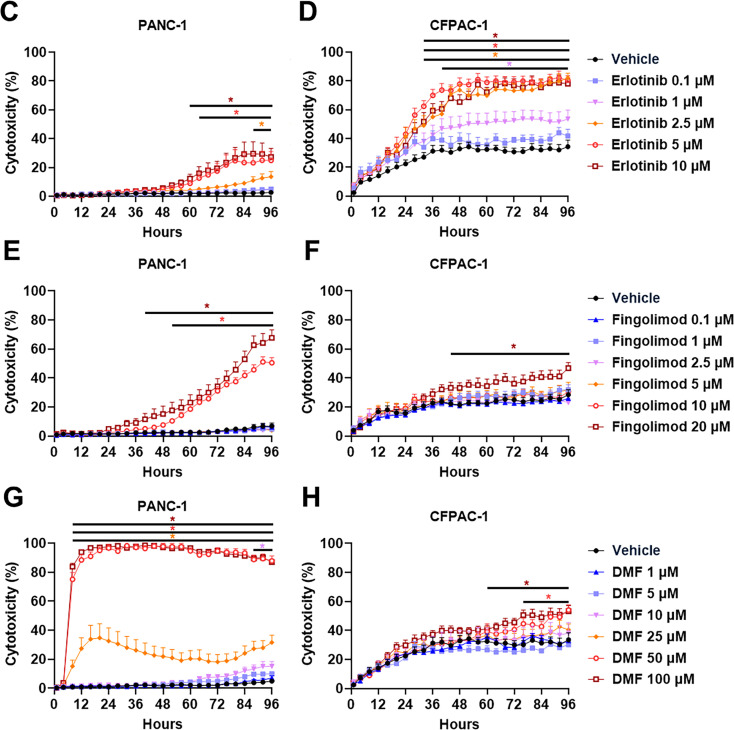
Effect of Gemcitabine, Erlotinib, Fingolimod, and Dimethyl fumarate (DMF) on pancreatic cancer cells in 3D tumor spheroid assay. (**A**,**B**) Dose-response effect of Gemcitabine on PANC-1 (**A**) and CFPAC-1 (**B**) spheroid cytotoxicity. (**C**,**D**) Dose-response effect of Erlotinib on PANC-1 (**C**) and CFPAC-1 (**D**) spheroid cytotoxicity. (**E**,**F**) Dose-response effect of Fingolimod on PANC-1 (**E**) and CFPAC-1 (**F**) spheroid cytotoxicity. (**G**,**H**) Dose-response effect of DMF on PANC-1 (**G**) and CFPAC-1 (**H**) spheroid cytotoxicity. The assay was done with 3 to 4 replicates from 3 independent experiments. Data represents mean and SEM. Statistical differences were determined using a mixed-effects model (REML, groups and time as factors) and Bonferroni’s multiple comparisons test. Statistical significance vs. vehicle is indicated by colored asterisks; each colored asterisks correspond to one specific concentration; *p < 0.05

We treated preformedPANC-1 and CFPAC-1 spheroids with Fingolimod and DMF, and compared their effects to those of Gemcitabine and Erlotinib over a 96-h period. We confirmed that Gemcitabine exhibits a significant dose-dependent cytotoxic effect in PANC-1 spheroids (Fig. 2A) and a significant cytotoxic effect in CFPAC-1 spheroids regardless of the dose (Fig. 2B). Erlotinib induced significant dose-dependent cytotoxicity in both PANC-1 and CFPAC-1 spheroids (Fig. 2C,D). These two reference molecules have therefore allowed us to show that spheroid model accurately mimic the tumor response to treatment. For Fingolimod the two highest doses resulted in a significant cytotoxic effect in PANC-1 spheroids (Fig. 2E), while only the highest dose of 20 µM led to a significant but modest cytotoxic effect in CFPAC-1 spheroids (Fig. 2F). DMF induced significant cytotoxicity in PANC-1 spheroids (Fig. 2G). In CFPAC-1 spheroids, only the two highest doses of 50 and 100 µM of DMF resulted in a significant cytotoxic effect (Fig. 2H). Overall, Fingolimod and DMF displayed toxicity comparable to the standards of care, Gemcitabine, or Erlotinib, in these cellular models.

### 3.3 Fingolimod and DMF Delayed Human PC Tumor Growth In Vivo in Xenograft Mouse Models

**Figure 3:**
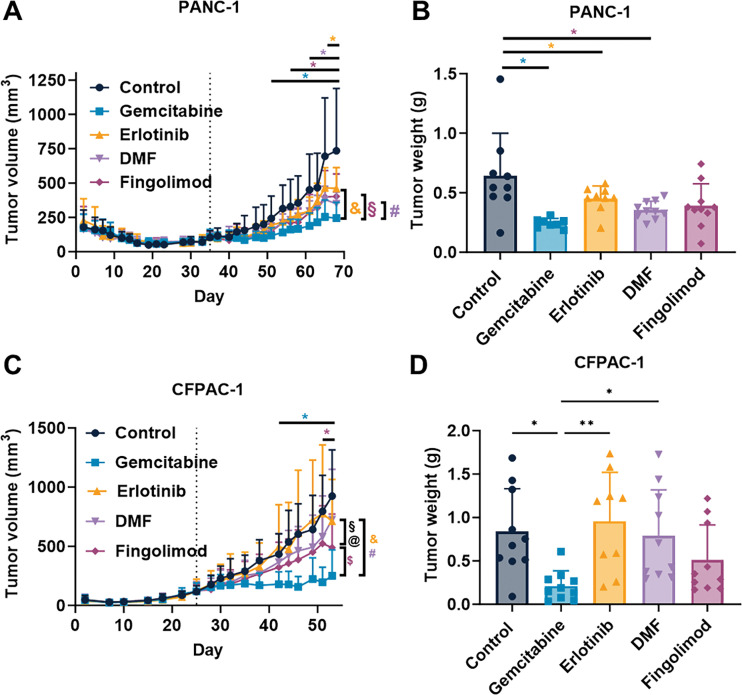

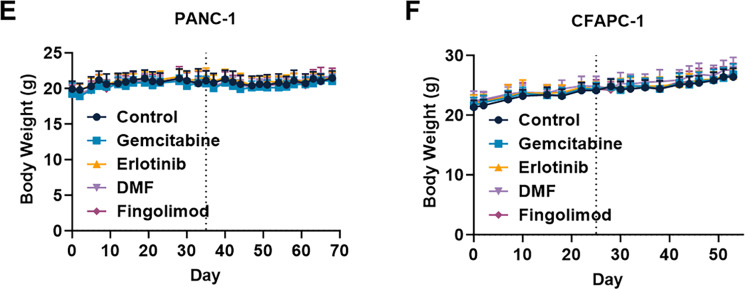
Effect of treatments in mouse pancreatic cancer model. (**A**,**C**) Impact of Gemcitabine at 80 mg/kg administrated once a week (i.p.), Erlotinib at 50 mg/kg administrated 5 times a week (p.o.), DMF at 30 mg/kg administrated 5 times a week (p.o.), and Fingolimod at 5 mg/kg administrated 5 times a week (p.o.) on tumor volume in PANC-1 xenograft (**A**) and CFPAC-1 xenograft (**C**) mouse models. (**B**,**D**) Impact of treatments on tumor weight collected after euthanasia in PANC-1 xenograft (**B**) and CFPAC-1 xenograft (**D**) mouse models. (**E**,**F**) Effects of treatments on mouse body weight in PANC-1 xenograft (**E**) and CFPAC-1 xenograft (**F**) mouse models. Discontinuous vertical line highlights treatment beginning. Data represents mean and SD. Statistical differences between the groups for the tumor volume and body weight were determined using a mixed-effects model (groups and time as factor) followed by Tukey’s multiple comparisons test (*p < 0.05 treatment vs. control, ^&^*p* < 0.05 Gemcitabine vs. Erlotinib, ^#^*p* < 0.05 Gemcitabine vs. DMF, ^§^*p* < 0.05 Fingolimod vs. Erlotinib, ^$^*p* < 0.05 Gemcitabine vs. Fingolimod, ^@^*p* < 0.05 Fingolimod vs. DMF). Statistical differences between tumor weight were determined using a one-way ANOVA followed by Tukey’s multiple comparisons test (*p < 0.05 and **p < 0.01). n = 10 mice/group at the beginning of treatments

In our study, we used two xenograft mouse models to assess the possible effect of Fingolimod and DMF on the progression of PC *in vivo* compared to Gemcitabine and Erlotinib. The results demonstrated that all four therapeutic interventions significantly suppressed tumor growth in the PANC-1 xenograft model, starting to be significant from day 51 for Gemcitabine, day 58 for Fingolimod, day 61 for DMF and from day 68 for Erlotinib (Fig. 3A). Gemcitabine-treated mice exhibited the most pronounced inhibition of tumor growth (Fig. 3A). The tumor weight collected after mouse sacrifice also displayed a reduction which was significant for all treatments except Fingolimod (Fig. 3B). In the CFPAC-1 xenograft model, we demonstrated that Gemcitabine and Fingolimod significantly repressed tumor growth from day 43 and day 53, respectively while DMF and Erlotinib did not (Fig. 3C). Again, Gemcitabine led to higher inhibition of tumor growth (Fig. 3C). Gemcitabine also induced a significant decrease of tumor weight as compared to control-treated mice, while Fingolimod tended to decrease it (Fig. 3D).

We also assessed mouse body weight and behavior in accordance with the ethical parameters. Notably, no significant changes in body weight were observed across treatment groups in either model (Fig. 3E,F). Markedly, no death or alteration in mouse behavior were seen in the groups treated with Fingolimod and DMF (including normal temperature, absence of dyspnea, normal consumption of food and water, absence of balance issues, and absence of sedation). Collectively, our findings indicate that both Fingolimod and DMF exhibited a favorable tolerance profile in murine subjects.

## 4 Discussion

Our work revealed that DMF and Fingolimod can significantly impede the progression of pancreatic tumors in *in vitro* and *in vivo* experiments. Indeed, Fingolimod is described to be particularly effective in reducing tumor volume relative to vehicle-treated mice across various tumor models [40]. DMF has also demonstrated anti-tumor effects in various tumor models, suggesting that both DMF and Fingolimod may be potential candidates for targeting pancreatic cancer [42]. We focused on pancreatic tumor cells to evaluate their responses to these treatments in 3D culture conditions. Notably, our findings demonstrated that Fingolimod and DMF triggered different cellular responses *in vitro*. While DMF directly induced cytotoxicity in tumor spheroids, Fingolimod did not produce a direct effect on cancer cells *in vitro*. Fingolimod and DMF were compared to two standards of care used to treat PC patients, Gemcitabine and Erlotinib. Gemcitabine exhibited a clear anti-tumor effect in PANC-1 and CFPAC-1 *in vitro* and *in vivo* models, superior to that observed with Fingolimod or DMF. Conversely, Erlotinib did not demonstrate an improved anti-tumor effect compared to both Fingolimod and DMF; in fact, the effects of Fingolimod and DMF were even more pronounced in the CFPAC-1 *in vivo* model. Altogether, our data offer the first proof of concept demonstrating the potential of DMF in 3D *in vitro* and *in vivo* pancreatic cancer models and complementary evidence of Fingolimod anticancer effects. We believe that this work will encourage further preclinical studies to evaluate the mechanism of action of Fingolimod and DMF as anti-cancer drugs.

Data from the literature emphasize the potential relevance of Fingolimod as an inhibitor of S1P signaling and DMF as a modulator of the NRF2 pathway for targeting pancreatic cancer in patients. Indeed, S1P acts as a pleiotropic lipid mediator regulating multiple signaling pathways. S1P is involved in cancer progression through the activation of pro-survival signaling, invasion, tumor angiogenesis, and cell metabolism, which are hallmarks of cancer that contribute to patient death [40,47]. S1P levels are elevated in tumor tissues compared to non-tumoral tissues [48]. Moreover, high expression of the phosphorylated form of sphingosine kinase 1, one of the principal enzymes that phosphorylates sphingosine into S1P, is correlated with high tumor invasion and worse disease-specific survival in PDAC patients [49]. Fingolimod has been shown to inhibit sphingosine kinase-1 activity or promote its degradation [40]. On the other hand, NRF2, as a master regulator of redox homeostasis, influences the fate of pancreatic tumor lesions and cancer progression [42]. NRF2 is highly upregulated in human pancreatic tumors, and its nuclear expression is associated with poor survival [50,51]. NRF2 is also correlated with tumor stage [52]. Our work additionally demonstrated that S1PR1, S1PR2, S1PR3, S1PR4, and NRF2 are significantly upregulated in pancreatic tumors when compared to non-tumoral tissues, except for S1PR5, which does not show similar changes (Fig. 1). Given the involvement of the S1P-S1PRs and NRF2 axes in PC, modulating these pathways with agents like Fingolimod and DMF might offer therapeutic benefits (reviewed in [27,53]). Fingolimod could act on S1PRs but also on an alternative mechanisms of action that rely on the modulation of sphingosine kinase-1 directly, which could both influence tumor progression in PC [40]. Alternatively, DMF, at high doses, inhibits NRF2 in cancer models [42].

Both Fingolimod and DMF exhibited anti-tumor properties in our experimental models. Interestingly, the administration of these agents showed favorable tolerability *in vivo* (e.g., absence of significant body weight changes, normal temperature, absence of dyspnea, normal consumption of food and water, absence of balance issues, and absence of sedation). Moreover, in individuals diagnosed with multiple sclerosis, Fingolimod and DMF have been associated with a commendable safety profile, characterized by manageable adverse effects [11,12]. The doses of Fingolimod and DMF used in this study were based on both non-toxic *in vivo* doses and relevant clinical doses with good safety. Indeed, data from the literature demonstrated that a dose equivalent to 30 mg per day for Fingolimod and 200 mg per day for DMF displays positive safety clinical profiles [54–56]. Fingolimod and DMF exhibit minimal or positive effects on normal cells [42,53]. Conversely, standard chemotherapies such as Gemcitabine or even targeted therapies such as Erlotinib induced cell death in normal cells at equivalent clinical doses [8,57]. The clinical efficacy of Gemcitabine in PC is often limited by resistance and systemic toxicity [8]. We observed that Gemcitabine significantly decreased tumor growth in our mouse models of PC, corroborating existing literature [58,59]. We established that Fingolimod produced anti-cancer effects in both xenograft models, and DMF was effective in the PANC-1 xenograft model, all while avoiding the toxicities commonly linked to chemotherapy. Although Erlotinib offers improved safety compared to chemotherapeutic drugs such as Gemcitabine, it can still result in skin and liver toxicities, which may compromise the quality of life for patients and could lead to the discontinuation of therapy [8]. Alternative therapies with a low toxicological profile are still mandatory for patients. We observed that Gemcitabine demonstrated a reduction in tumor growth in our xenograft models, consistent with previous reports [8]. Notably, both Fingolimod and DMF exhibited comparable or even higher anti-tumor efficacy as compared to Erlotinib, and reached partially the effect of Gemcitabine, possibly without the associated chemotherapy-induced toxicity in humans (Fig. 3). While targeted therapies, like Erlotinib, offer improved safety profiles relative to traditional chemotherapeutic agents, they are not devoid of adverse effects, including skin toxicity and hepatotoxicity, which can impair patient quality of life and necessitate treatment discontinuation [57]. Given the substantial toxicity profile, Fingolimod and DMF might present interesting translational interest, despite further investigation are still required. Altogether, the efficacy and safety data highlight the interest of Fingolimod and DMF as therapy for at least preclinical models of PC.

Our *in vitro* findings indicate that the effects of Fingolimod and DMF vary depending on the cell line. Indeed, each cell line has its own genetic background, which influences its response to treatment, and this helps explain the differences in patient response. Fingolimod induces apoptosis of PANC-1, AsPC-1, and BxPC-3 cells in 2D cultures [21]. DMF also showed dose-dependent anti-tumor effects in 2D cultures with PANC-1 and CFPAC-1 lines [33]. The results obtained on 3D cultures of spheroids confirmed the anti-tumor activity on more complex *in vitro* PC models (Fig. 2). We observed an anti-proliferative effect for both Fingolimod and DMF in our xenograft models with PANC-1 and CFPAC-1 cells. Both molecules showed *in vivo* effects as tumor growth inhibitors and did not appear to present general toxicity. These results corroborate studies carried out on triple-negative breast cancer and on lung cancer in which Fingolimod and DMF also presented an anti-tumor effect *in vivo* without general toxicity [14,15]. Combining our spheroid model data with literature, we conclude that Fingolimod and DMF directly impact tumor cell viability [27,40].

However, both molecules also affect other cell types within the TME. Inflammation and the development of an immunosuppressive microenvironment play a role in the development and progression of PC [6]. Fingolimod sequesters lymphocytes in the lymph nodes [13]. Similarly, DMF has anti-inflammatory and immunomodulatory capacities, inducing a reduction of the peripheral number of mature B and T cell subtypes, limiting their activity in the central nervous system [60]. However, this effect could be detrimental in PC by also reducing cytotoxic T cell numbers, which are critical for anti-cancer responses [61]. While both drugs exhibit immunomodulatory effects in multiple sclerosis, the immunomodulatory role in cancer remains less clear. The impact of DMF on immune function in cancer is particularly ambiguous with studies reporting opposing effects depending on the cancer type. Jiang and collaborators demonstrated that DMF enhanced CD8+ T cell infiltration in a syngeneic model of cervical cancer, contributing to an anti-tumor response; notably, this effect was absent in immunodeficient (lacking functional T cells) nude mice [62]. Conversely, Gao and collaborators suggested that DMF may facilitate immune escape in a syngeneic renal cancer model, potentially promoting cancer progression [63].

In contrast, the immunomodulatory effects of Fingolimod in cancer have been more thoroughly investigated. Spranger and collaborators demonstrated that combining Fingolimod with an immune checkpoint inhibitor in a syngeneic melanoma model using B16F10 cells did not impair efficacy and even enhanced anti-tumoral effect. They also observed that despite a reduction of CD3 positive cells in peripheral blood, T cell infiltration within the tumor tissue was not affected [64]. In addition, Ng and collaborators reported that Fingolimod did not alter CD3+ cell infiltration into the tumor tissue but blocked tumor progression in immunocompetent rat liver tumor model [65]. Several studies have also reported anti-tumor effects of Fingolimod in xenograft model lacking functional T cells (reviewed in [40,53]). In our published work, we observed a direct cytotoxic effect of both Fingolimod and DMF on tumor spheroids and *in vivo* in immunodeficient and immunocompetent mice [15]. Furthermore, we demonstrated that Fingolimod repressed tumor growth in both syngeneic and xenograft models of breast cancer [14]. Collectively, these data suggest that Fingolimod anti-tumor efficacy is only modestly influenced by immune system modulation, whereas the role of DMF remains less clear. In both cases, we observed a direct effect on tumor cells.

Another potential benefit of Fingolimod and DMF in PC is their ability to enhance the efficacy of existing treatments and overcome resistance mechanisms, such as those observed with Gemcitabine [4]. Resistance to Gemcitabine has been linked to the activation of the nuclear transcription factor kappa B (NF-κB) pathway [66]. Numerous studies demonstrated the key role of NF-κB in the development of cancers (summarized in [67]). Furthermore, previous study reports that Fingolimod would be a potent inhibitor of interactions between NF-κB and STAT3 signaling pathways [32]. On the other hand, DMF has also already shown its effects as an NF-κB inhibitor both *in vitro* and *in vivo* on cutaneous T-cell lymphoma, melanoma, and breast cancer [68–71]. Both DMF and Fingolimod have demonstrated the ability to sensitize cancer cells to chemotherapy in other tumor types (reviewed in [27,40]), suggesting a possible role in overcoming resistance mechanisms in PC. Fingolimod enhanced tumor growth reduction in mouse xenograft models when combined with Gemcitabine, without superior monotherapy effects as we observed in our work [32]. However, we also already evaluated the effect of combination therapies in one syngeneic cancer model of breast cancer and combination treatment did not enhance the maximal therapeutic effect that do not indicate any synergistic effect [14]. Thus, the effect depend on tumor type, highlighting the need to evaluate both monotherapy and combination therapy.

In the present work, we observed a decrease of tumor growth upon both Fingolimod and DMF treatments in PANC-1 xenograft model and upon Fingolimod only in CFPAC-1 xenograft model. In 3D models, both molecules induced cytotoxicity. However, discrepancies were noted between the *in vitro* and *in vivo* results. It is possible that the *in vivo* drug concentration in CFPAC-1 tumors did not reach or sustain an effective level observed for cytotoxicity *in vitro*. However, we already identified that higher dose of DMF was toxic for mice [15]. Moreover, the CFPAC-1 spheroid model may not fully recapitulate the complexity of the *in vivo* tumor, including vascularization or intrinsic resistance mechanism. Interestingly, Grattarola and collaborators demonstrated that CFPAC-1 cells display a low level of NRF2 as compared to PANC-1 cells [72]. Since NRF2 is a direct described target of DMF and is involved in cancer progression [27], we can expect that CFPAC-1 might be less sensitive to DMF therapy as compared to Fingolimod. Given its limited direct impact on tumor cells, DMF is likely to influence alternative targets within the TME. Notably, DMF has the potential to modulate stromal cells within the TME, which are crucial in tumor progression [27]. The effects of Fingolimod on tumor morphology and microenvironment are important, as it facilitates tissue remodeling, promotes the normalization of blood vessels, and leads to a reduction in hypoxia [73]. Both Fingolimod and DMF have demonstrated anti-angiogenic activity *in vitro* and *in vivo* [32,74]. Angiogenesis, or the formation of blood vessels from pre-existing vessels, is described as an essential process for tumor progression, thus targeting it could also provide an additional indirect impact on tumor growth [75,76]. Thus, the modulation of tumor response is influenced by the TME, which may help explain the varying therapeutic responses observed in our models, suggesting that Fingolimod and DMF operate through distinct mechanisms.

Nevertheless, this study acknowledges several limitations. While our study provides both *in vitro* and *in vivo* characterization of the phenotypic responses upon Fingolimod and DMF, a notable limitation is the absence of molecular mechanism analysis. S1PR inhibition by Fingolimod and NRF2 activation by DMF in other cancer types contribute to the anti-tumor response (review in [40,53]). The precise mechanism by which Fingolimod and DMF participates in the modulation of tumor response signaling pathways remains to be explored. Moreover, future studies should evaluate the effect of combined treatments such as Gemcitabine plus Fingolimod or Gemcitabine plus DMF, compared to Gemcitabine alone, to assess potential synergistic or additive effects or to confirm the absence of adverse interactions. Our xenograft mouse models did not allow us to analyze the impact of the immune system in response to treatments but were used to confirm an independent anti-cancer action. Ultimately, a more comprehensive characterization of immune system and pancreatic TME involvement, particularly using reconstituted human immune system mouse models xenografted orthotopically with human pancreatic cancer cells into the pancreas would be justified and will be evaluated in a future work.

## 5 Conclusion

In conclusion, we validated the first evaluation of DMF efficacy *in vivo* and in 3D complex *in vitro* models and provided complementary evidence of Fingolimod anti-cancer effects in two independent animal models of PC. Moreover, we demonstrated that both Fingolimod and DMF can exert direct cytotoxic effect *in vitro* using 3D spheroid culture of PC cell lines. This work provides preclinical evidence that supports the potential therapeutic benefits of Fingolimod and DMF. Although the effects of Fingolimod and DMF did not surpass those of Gemcitabine and Erlotinib, these findings suggest that Fingolimod and DMF presented a good safety profile may be considered for further preclinical exploration. Overall, our data suggest that Fingolimod and DMF are worthy of further PC preclinical and translational assessments.

## Supplementary Materials



## Data Availability

The raw data supporting the findings of this study are available from the corresponding author, Dr. Tristan Rupp, upon reasonable request.

## Supplementary Materials

The supplementary material is available online at https://www.techscience.com/doi/10.32604/or.2025.072141/s1.

## Abbreviations

5-FU: 5-fluorouracil
AAALAC: Association for Assessment and Accreditation of Laboratory Animal Care
DMF: Dimethyl fumarate
DMSO: Dimethyl sulfoxide
EGFR: Epidermal growth factor receptor
GTEx: Genotype tissue expression
i.p.: Intraperitoneal
NF-κB: Nuclear transcription factor kappa B
NRF2: Nuclear factor erythroid 2-related factor 2
OS: Overall survival
PBS: Phosphate-buffered saline
PAAD: Pancreatic adenocarcinoma
PC: Pancreatic cancer
PDAC: Pancreatic ductal adenocarcinoma
PEG: Polyethylene glycol
p.o.: Per os
REML: restricted maximum likelihood
S1P: Sphingosine-1-phosphate
S1PR: Sphingosine-1-phosphate receptor
s.c.: Subcutaneous
SD: Standard deviation
SEM: Standard error to the mean
TCGA: The cancer genome atlas
TME: Tumor microenvironment
ULA: Ultra low adhesive
