# Supplementary material for: Therapeutic Potential of Fingolimod and Dimethyl Fumarate in Preclinical Pancreatic Cancer Models
Source: Oncol Res. 2026 Feb 24;34(3):12. doi: 10.32604/or.2025.072141 (PMC12963673; doi:10.32604/or.2025.072141)
Supplement: Supplementary file 1 [file OncolRes-34-72141-s001.docx]

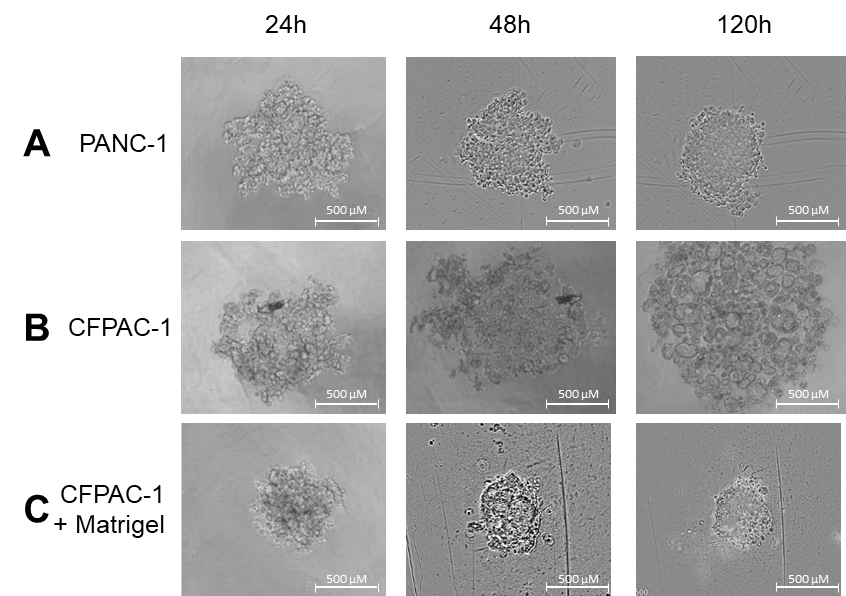


**Figure S1: Evaluation of spheroid formation using PANC-1 and CFPAC-1 cells**. Representative pictures of **(A)** PANC-1 cells, **(B)** CFPAC-1 cells, and **(C)** CFPAC-1 cells supplemented with Matrigel® after 24, 48, and 120 hours of culture in 96-well CellCarrier Spheroid Ultra Low Adhesive (ULA) Microplates™. Scale = 500 µm.
